# Genome-wide identification and characterization of lncRNAs in sunflower endosperm

**DOI:** 10.1186/s12870-022-03882-5

**Published:** 2022-10-22

**Authors:** Shuai Yu, Zhichao Zhang, Jing Li, Yanbin Zhu, Yanzhe Yin, Xiaoyu Zhang, Yuxin Dai, Ao Zhang, Cong Li, Yanshu Zhu, Jinjuan Fan, Yanye Ruan, Xiaomei Dong

**Affiliations:** 1 College of Bioscience and Biotechnology, Shenyang Agricultural University, Shenyang, 110866 Liaoning China; 2 Shenyang City Key Laboratory of Maize Genomic Selection Breeding, Shenyang, 110866 Liaoning China; 3 School of Traditional Chinese Materia Medica, Shenyang Pharmaceutical University, Shenyang, China; 4 State Key Laboratory of Maize Bio-Breeding, Shenyang, China; 5 State Key Laboratory of the Northeast Crop Genetics and Breeding, Shenyang, China

**Keywords:** Sunflower, Endosperm, Long non-coding RNAs, DNA methylation, Genomic imprinting

## Abstract

**Background:**

Long non-coding RNAs (lncRNAs), as important regulators, play important roles in plant growth and development. The expression and epigenetic regulation of lncRNAs remain uncharacterized generally in plant seeds, especially in the transient endosperm of the dicotyledons.

**Results:**

In this study, we identified 11,840 candidate lncRNAs in 12 day-after-pollination sunflower endosperm by analyzing RNA-seq data. These lncRNAs were evenly distributed in all chromosomes and had specific features that were distinct from mRNAs including tissue-specificity expression, shorter and fewer exons. By GO analysis of protein coding genes showing strong correlation with the lncRNAs, we revealed that these lncRNAs potential function in many biological processes of seed development. Additionally, genome-wide DNA methylation analyses revealed that the level of DNA methylation at the transcription start sites was negatively correlated with gene expression levels in lncRNAs. Finally, 36 imprinted lncRNAs were identified including 32 maternally expressed lncRNAs and four paternally expressed lncRNAs. In CG and CHG context, DNA methylation levels of imprinted lncRNAs in the upstream and gene body regions were slightly lower in the endosperm than that in embryo tissues, which indicated that the maternal demethylation potentially induce the paternally bias expression of imprinted lncRNAs in sunflower endosperm.

**Conclusion:**

Our findings not only identified and characterized lncRNAs on a genome-wide scale in the development of sunflower endosperm, but also provide novel insights into the parental effects and epigenetic regulation of lncRNAs in dicotyledonous seeds.

**Supplementary Information:**

The online version contains supplementary material available at 10.1186/s12870-022-03882-5.

## Background

In eukaryotic, approximately 90% of the whole genomes are transcribed into RNA [1]. Among these transcripts, only ~ 2% of them can be translated into proteins, and majority of them are defined as non-coding RNAs (ncRNAs) [2, 3]. The ncRNAs are functional RNA molecules that do not encode proteins and possess key regulatory functions [4]. According to their functions, ncRNAs can be divided into housekeeping ncRNAs and regulatory ncRNAs [5]. Long non-coding RNAs (lncRNAs) are an important group of regulatory ncRNAs that are longer than 200 nucleotides [6]. According to their genomic positions, lncRNAs can be classified into long intervening noncoding RNA (lincRNA), antisense lncRNA (lncNAT), intron lncRNA, and sense lncRNA [7, 8]. Compared to protein-coding genes (PCgenes), most lncRNAs exhibit lower conservation across species, lower expression levels and strong tissue-specific expression [9–14]. In plants, more and more studies have shown that lncRNA plays a critical role in many biological processes, including development processes, reproduction processes and stress responses [15–18].

With the rapid development of high-throughput RNA sequencing, thousands of lncRNAs have been identified and characterized in several plants [10, 13, 14, 19–25]. Although only a few lncRNAs have known functions in current study, the functions and regulatory mechanisms of lncRNAs are diverse and complex [26–28]. For example, a NAT-lncRNA MAS can be induced by cold and activate of sense gene MADS AFFECTING FLOWERING4 (MAF4) for suppression of precocious flowering [29]. GARR2 can influence the plant height ideotype by involving in the modulation of the GA response in maize [30]. In addition, some lncRNAs also play pivotal roles in biotic and abiotic stress responses in plants. Enhanced expression of ALEX1 can activate the expression of jasmonic acid signaling pathway related genes in rice, and significantly improve rice resistance to *Xanthomonas oryzae* [31]. All in all, lncRNAs might play important biological roles during plant growth and development.

In recent years, the lncRNAs from seeds had been identified in many plants, including maize [32–34], *Brassica napus* [35], tree peony [36], castor bean [22], pigeonpea [37], *Ginkgo biloba* [38], and rice [39]. These lncRNAs might play a complex regulatory role in seed development. In *Brassica napus* and tree peony developing seeds, lncRNAs probably have effect on lipid metabolism [35, 36]. In maize and castor bean, lncRNA might play a part in regulating endosperm development by genomic imprinting [22, 33]. In plants, endosperm is a triploid tissue with a 2:1 maternal:paternal genome ratio [40]. Genomic imprinting, mainly occurring in endosperm, refers to allele-specific expression of genes depending on parental origin [41, 42]. So far, imprinted long noncoding RNAs were identified in endosperm of several plants [22, 33, 43]. Recently, a maternally expressed lncRNA MISSEN were reported as a regulator to modulate rice endosperm development [44]. In flowering plants, seed development is an intricate and ordered process that is regulated by both genetic and epigenetic factors [45]. DNA methylation, a heritable epigenetic mark, can affect gene transcription and influence development [46–49]. Understanding the regulation of DNA methylation requires consideration of the distribution of methylation across the gene and lncRNA. Hence, acquisition of lncRNAs and its DNA methylation pattern in sunflower endosperm will lay a solid foundation for further exploration its influence on seed development.

Sunflower (*Helianthus annus* L.) is the fourth most important oil crop in the world [50]. And the endosperm was easily separated from the embryo and other maternal tissues, which avoid surrounding tissue contamination. In this study, we analyzed RNA sequencing (RNA-seq) and DNA methylation data, and comprehensively characterized the genomic expression, DNA methylation and inheritance patterns of lncRNAs in endosperm tissues of sunflower. Together, our findings will be helpful for further research on the potential functions, parental effects and epigenetic regulation of lncRNAs in flowering plants.

## Results

### RNA sequencing and identification of lncRNAs in sunflower endosperm

**Fig. 1:**
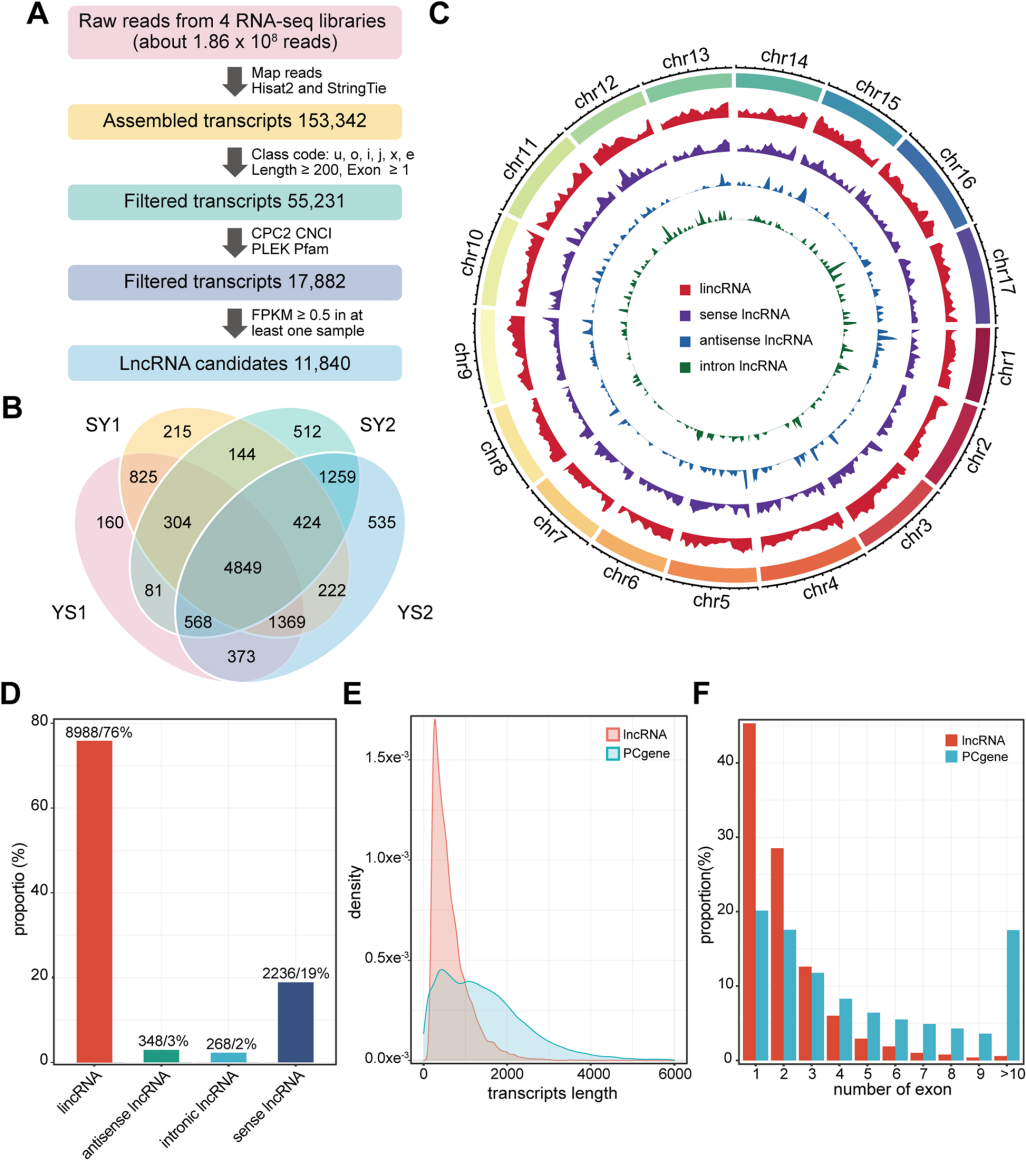
Identification and characterization of long non-coding RNAs (lncRNAs) in sunflower endosperm at 12 DAP. **A** Schematic pipeline for the identification of lncRNAs in sunflower endosperm; **B** Expressed lncRNAs in two crosses. Venn diagrams showing the number of common and specific lncRNAs in the four libraries; **C** Distribution of the lincRNA (red), sense lncRNA (purple), antisense lncRNA (blue) and intron lncRNA (green) on each chromosome; **D** Classification of total identified lncRNAs including lincRNA, antisense-lncRNA, intronic-lncRNA and sense-lncRNA; **E** Length density distributions of long non-coding RNAs (lncRNAs) and protein-coding genes (PCgenes); **F** Distribution of exon numbers in lncRNAs and PCgenes

In order to explore the characteristics of lncRNA expression in sunflower endosperm, the RNA-seq data of 12 days after pollination (DAP) endosperm tissue from reciprocal hybrid pairs of our previously published was performed to identify lncRNA [51]. About 45 million clean reads were acquired from each of the four libraries [SY1(138A × 398B), YS1(398A × 138B), SY2(723A × 6B), YS2(6A × 723B)] for further analysis (Additional file 1: Table S1). After reassembling and mapping, between 88.9 and 91.06% of the reads were successfully aligned to the sunflower genome (Additional file 1: Table S1). Then, the mapped clean reads were assembled as a transcript using StringTie, and we identified 153,342 transcripts (Fig. 1a). Subsequently, the transcripts were filtered based on their type of transcripts and sequence length (less than 200 nucleotides), and 55,231 transcripts were retained (Fig. 1a). Next, the protein-coding potential of remaining transcripts were predicted jointly by four analyses: CPC2 analysis (Coding Potential Calculator), CNCI analysis (Coding-Non-Coding Index), PLEK analysis (predictor of long non-coding RNAs and messenger RNAs based on an improved k-mer scheme) and Pfam protein domain analysis. After the four computational approaches prediction, 17,882 transcripts were obtained (Fig. 1a). Finally, we obtained 11,840 transcripts as putative lncRNAs by expression level [fragments per kilobase of transcript per million mapped reads (FPKM) ≥ 0.5] in sunflower endosperm (Fig. 1a). Thereinto, 11,840 lncRNAs were identified in all tissues (Additional file 2: Table S2), including 9534 and 10,640 lncRNAs in SY1/YS1 and SY2/YS2, respectively (Fig. 1b).

In order to explore the potential functions of lncRNAs, we defined co-expressed protein-coding genes (PCgenes) that located within 100 kb from all candidate lncRNAs. The functional annotation of these PCgenes were carried out by assignment of GO terms. There were 13 biological processes including “hormone-mediated signaling pathway”, “response to abscisic acid”, “response to lipid” and so on, and 11 molecular functions including “hormone binding”, “carboxylic acid binding” and so on (Additional file 3: Table S3).

We examined the overlap of the lncRNA transcripts in the four sunflower F1 hybrid endosperm. As shown, about 88.0% (7347) of the lncRNAs with a genome hit showed evidence of expression in SY1 and YS1 endosperm, and about 87.2% (7100) of the lncRNAs with a genome hit showed evidence of expression in SY2 and YS2 endosperm (Fig. 1b). But only half of (4849) lncRNAs were found in both of two crosses (Fig. 1b), which indicated that lncRNAs tend to be specific expression in intraspecies.

### The genomic characteristics of lncRNAs in sunflower endosperm

Using the circus program, these lncRNAs were mapped to the 17 chromosomes of the sunflower genome, and we found that these lncRNAs were evenly distributed in all chromosomes with no obvious location preference (Fig. 1c). Based on their locations in the genome, the 11,840 lncRNAs in sunflower endosperm were divided into four types: 8988 (76%) lincRNAs, 348 (3%) lncNATs, 268 (2%) intronic-lncRNAs, and 2236 (19%) sense-lncRNAs, respectively (Fig. 1d). The lncRNA identified in both of two crosses were tend to be located in genic region compared with all lncRNAs (Additional file 4: Fig. S1). To more clearly characterize the lncRNA in sunflower endosperm, the identified lncRNAs were performed through comparing with that of PCgenes. The sequence length of lncRNA transcripts (average length of 647 nt) was shorter than the PCgenes (average length of 1474 nt) (Fig. 1e). The number of exons of the lncRNAs was significantly lower than that of the PCgenes (Fig. 1f). Approximately 86% of lncRNAs with 1–3 exons were significantly higher proportion than PCgenes (49%). As the number of exons increased, the proportion of lncRNAs decreased.

### Association of the expression of lncRNAs and protein-coding genes

**Fig. 2:**
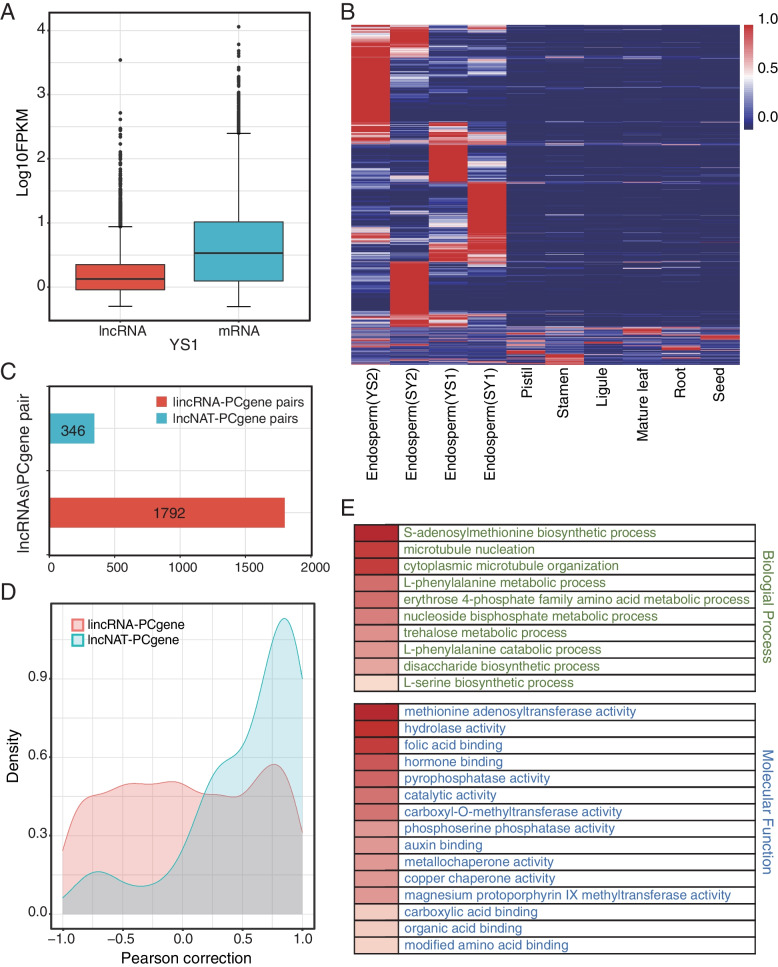
Expression of long non-coding RNAs (lncRNAs) and protein-coding genes (PCgenes). **A** Expression levels of lncRNAs and PCgenes in YS1 endosperm as illustrated by the boxplot; **B** The expression profile of lncRNAs among tissues; **C** Summary of various types and numbers of lncRNA–PCgene pairs in sunflower endosperm; **D** The density distribution of the Pearson correlation coefficient for lincRNA–PCgene and lncNAT–PCgene pairs; **E** A heat map showing the enrichment of GO terms in the biological process (BP) category and molecular function (MF). The colors of the heat map represents the *P*-value for each GO term value

The overall expression levels of lncRNAs were significantly lower than those of PCgenes in endosperm of two sunflower crosses (Fig. 2a, Additional file 5: Fig. S2). LncRNA have been found to show tissue-specific expression in plants [10, 13, 14, 22]. To explore the expression patterns of lncRNAs in sunflower, we downloaded and analyzed publicly available RNA-seq data sets of other sunflower tissues, including pistil, stamen, ligule, mature leaf, root, and seed. We found that most of the lncRNAs exhibited strong tissue-specific expression patterns in endosperm, and a small number of lncRNAs showed constitutive expression (Fig. 2b).

LncRNA affect gene expression in a cis (neighboring genes) or trans (distant genes) manner. To analyze the potential functions of these lncRNAs, we predicted the cis- and trans-target genes within 100 kb upstream and downstream of the lncRNAs. Pearson correlation coefficient (r_p_) was used to estimate the expression correlation of lncRNA-PCgene pairs. PCgenes with low expression levels (FPKM< 0.5) were removed. Accordingly, 1792 lincRNA-PCgene and 78 lncNAT-PCgene pairs were identified (Fig. 2c). We observed a high percentage of positive correlations (r_p_ ≥ 0.8, *P*-value < 0.01, t-test) in lincRNA-PCgene and lncNAT-PCgene pairs (Fig. 2d). The lncNAT-PCgenes pairs exhibited a stronger correlation than the lincRNA-PCgene pairs (Fig. 2d). A gene ontology analysis of those PCgenes showing strong correlation with the lncRNAs revealed that most lncRNAs were involved in methionine adenosyltransferase activity, auxin binding, carboxylic acid binding and so on (Fig. 2e).

### DNA methylation of lncRNAs

**Fig. 3:**
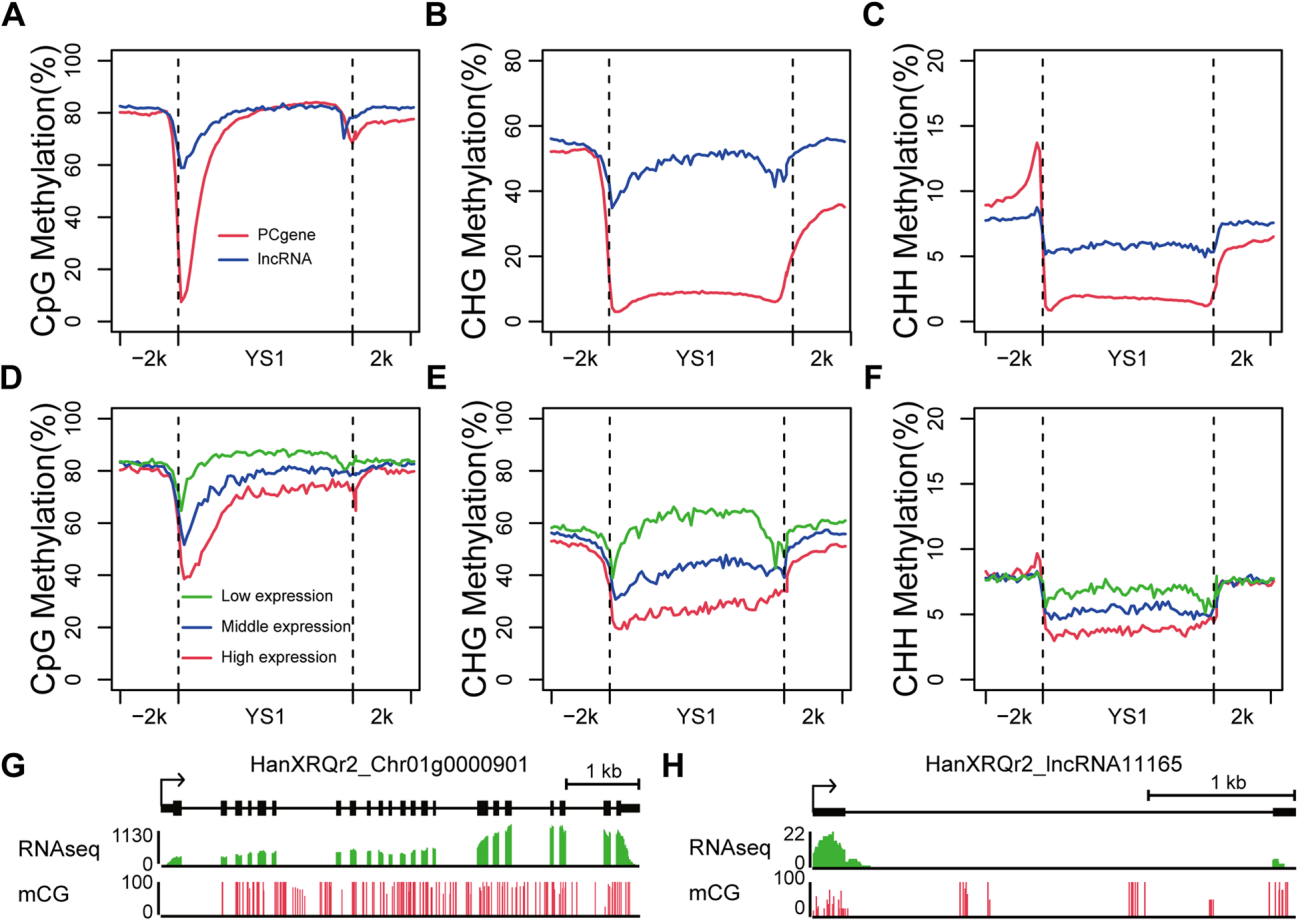
DNA methylation profiles of long non-coding RNAs (lncRNAs) and protein-coding genes (PCgenes) in sunflower endosperm. **A**-C Average DNA methylation levels of lncRNAs (blue lines) and PCgenes (red lines) in YS1 endosperm; **D**-**F** Association between DNA methylation and lncRNA expression in CG, CHG and CHH sequence contexts throughout the gene body and its 2-kb up- and downstream regions in YS1 endosperm. **G**, **H** Two examples of DNA methylation and gene expression at the PCgene (g) and lncRNA (f) were displayed, respectively. The expression level of transcribed regions is shown in green; The DNA methylation level of transcribed regions is shown in red

Since lncRNAs are important regulatory roles in many biological processes, their expression must be tightly regulated. The regulation by DNA methylation of the expression of PCgenes and lncRNAs has not been well characterized in sunflower. The overall methylation levels within the 2-kb flanking region and body region of both expressed PCgenes and lncRNAs (FPKM ≥0.5) was examined. In YS1 endosperm, the PCgenes and lncRNAs displayed a relatively lower methylation levels near the transcription start and stop sites in the CG context (Fig. 3a). The methylation levels of lncRNAs were significantly higher than PCgenes in transcription start sites. In the CHG context, the overall DNA methylation levels within the 2-kb flanking region and body region was substantially higher for lncRNAs (Fig. 3b). In the CHH context, for both lncRNAs and PCgenes, the level of DNA methylation was decreased near the transcription start sites (Fig. 3c). The overall DNA methylation levels of PCgenes in the upstream was higher than lncRNAs, whereas lncRNAs in downstream and gene body regions had a higher level of DNA methylation (Fig. 3c). Similarity, the overall methylation profiles of PCgenes and lncRNAs in SY1 endosperm was similar to those in YS1 endosperm (Additional file 6: Fig. S3).

To evaluate the relationship between DNA methylation level and expression levels of PCgenes and lncRNAs, we divided the PCgenes and lncRNAs into three groups according to their expression levels. The highly expressed lncRNAs displayed a relatively lower CG, CHG and CHH methylation levels at both their flanking and body regions (Fig. 3d-f). In contrast, the low expression level of lncRNAs had a higher methylation level for all three sequence contexts. The level of DNA methylation at the transcription start sites was negatively correlated with gene expression levels in lncRNAs. For example, areas near the TSS were about 40% methylation levels for the most highly expressed genes, but were nearly 70% methylation for the genes with lowest expression level. In the PCgenes, the results showed that mRNA transcript levels in endosperm were positively correlated to gene-body methylation levels, but were negatively significantly correlated to promoter methylation levels (Additional file 7: Fig. S4). In Fig. 3g and h, the integrated profiles of DNA methylation and gene expression at the HanXRQr2_Chr01g0000901 (PCgene) and HanXRQr2_lncRNA11165 (lncRNA) were displayed, respectively.

### Identification and characters of imprinted lncRNAs

Some lncRNAs exhibit allelic expression which is regulated by the parent-of-origin effects in endosperm of flowering plants. To systematically identify imprinted noncoding RNAs in sunflower endosperm. A total of 36 imprinted lncRNAs in sunflower endosperm were got (Additional file 8: Table S4). Among them, 32 are maternally expressed lncRNAs (MNC), whereas four are paternally expressed lncRNAs (PNC). Most of imprinted lncRNAs were located in intergenic region, including 30 intergenic lncRNAs, one intronic lncRNA, five sense lncRNA (Additional file 9: Fig. S5). These imprinted long noncoding transcripts have an average length of 1049 bp, ranging from 308 bp to 2711 bp (Additional file 8: Table S4), as estimated from regions covered by the sequencing reads.

**Fig. 4:**
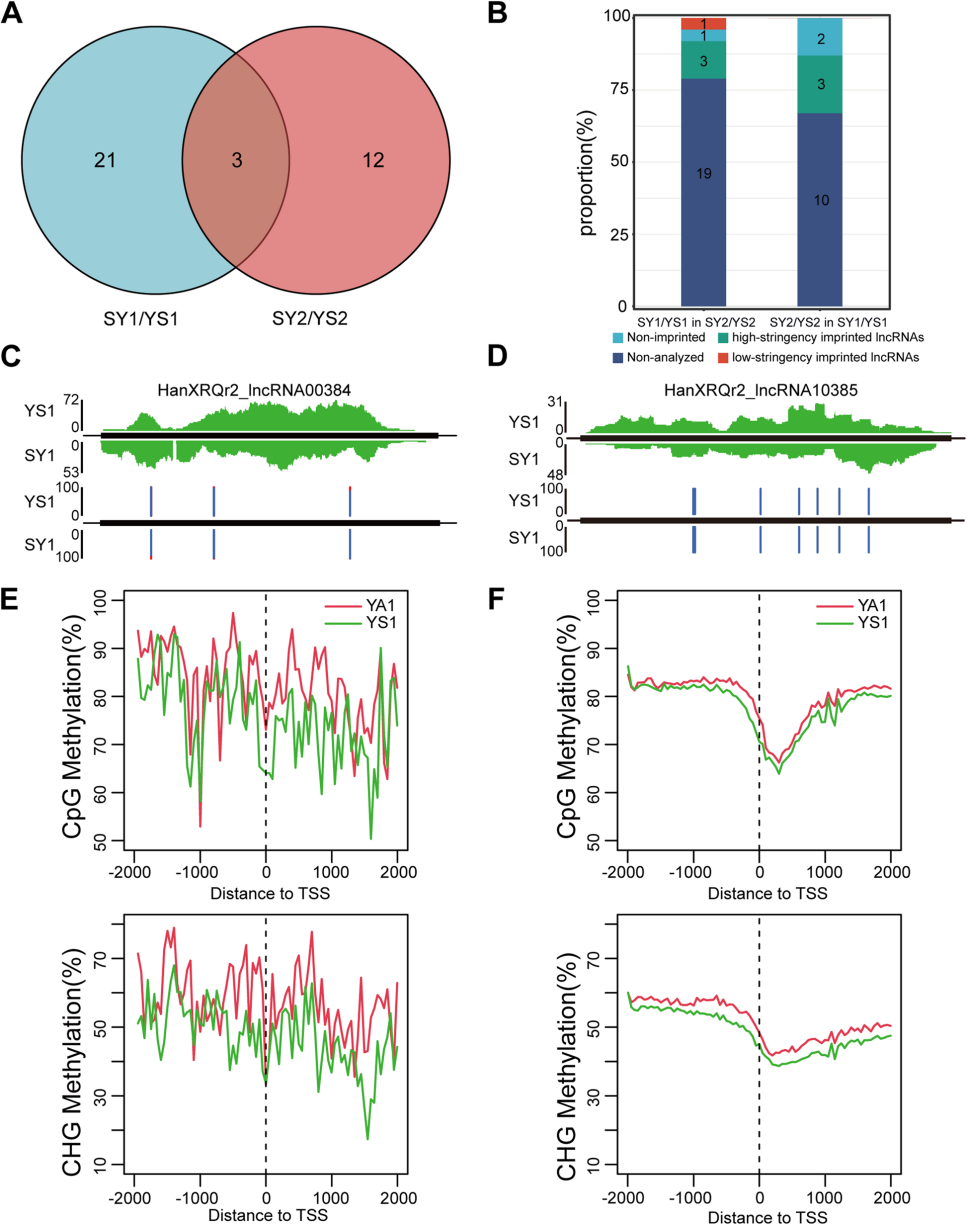
Identification of imprinted lncRNAs in sunflower endosperm at 12 DAP. **A** Venn diagram analysis of imprinted lncRNAs. The number of imprinted lncRNAs identified in two crosses are shown in the blue (SY1/YS1) and red (SY2/YS2) circles, respectively.; **B** Comparison of imprinted lncRNAs in two crosses of sunflower. Non-imprinted: lncRNAs not showing significant deviation from 2:1 ratio of maternal allele to paternal allele in each reciprocal hybrid. Non-analyzed: lncRNAs without sufficient read counts. Low-stringency imprinted lncRNA: lncRNAs showing significant deviation from 2:1 ratio of maternal allele to paternal allele in each reciprocal hybrid. High-stringency imprinted lncRNAs: lncRNAs in which favorable alleles were at least five times more than those of non-favorable alleles in both directions of a reciprocal cross; **C**, **D** Two examples of imprinted lncRNAs. The expression level of transcribed regions is shown in green for SY1 and YS1; The percentages of allelic reads of two imprinted lncRNAs for specific SNP sites are shown, with red lines for the paternal allele and blue lines for the maternal allele; Black rectangle, exon; black line, intron. **E**-**F** DNA methylation level distribution in imprinted lncRNAs (**E**) and all lncRNAs (**F**) around the transcription start site (TSS) region, including CG, CHG methylation

We assessed allelic imprinting variation in the two crosses (SY1/YS1 and SY2/YS2) as visualized in the Venn diagram (Fig. 4a). Although three (one MNC and two PNCs) imprinted lncRNAs were found to overlap in the two crosses, most of the imprinted lncRNAs identified in one cross tended to be imprinted in other reciprocal crosses (Fig. 4b). Imprinted lncRNAs found in only one set of reciprocal crosses usually lacked informative SNPs or had insufficient reads to identify if they were imprinted in other crosses (Fig. 4b). For example, among 24 imprinted lncRNAs (including 21 MNCs and three PNCs) identified in SY1/YS1 endosperm, four were MNCs/PNCs, one were non-imprinted gene and 19 (79.1%) had no polymorphisms or were not expressed in SY2/YS2 endosperm. Some of the examples of imprinted lncRNAs exhibited imprinting of alleles from some genotypes but not others. Figure 4c and d displays the expression profiles of two MNCs. As showed, all SNPs located at two MNCs exhibited significantly maternal bias.

Genomic imprinting is generally regulated by epigenetic modifications [52, 53]. The availability of DNA methylome data allowed us to investigate the relationship between DNA methylation and expression of the imprinted noncoding RNAs. In the CG and CHG context, the overall DNA methylation levels of the imprinted noncoding RNAs in the upstream 1 kb and gene body 5′ regions were slightly lower in endosperm than those in embryo (Fig. 4e, f).

### Identification of lncRNAs exhibiting allele-specific expression in cultivated sunflower lines for edible fruit and oil

LncRNAs exhibiting allele-specific expression (ASEG) may lead to phenotypic variation depending on the function of the genes. To better understand how parental alleles contribute to the development of endosperm, a genome-wide identification of lncRNAs exhibiting allele-specific expression were performed by comparing the read ratios of the parental alleles in RNA-sequencing data of hybrid endosperm. Consequently, the expression of 81 and 62 lncRNAs showed allelic bias toward cultivated lines for edible fruit (SA1 and SA2) and cultivated lines for oil (YA1 and YA2), respectively (Additional file 10: Table S5). Interestingly, lncRNAs showing allelic bias toward cultivated lines for edible fruit and cultivated lines for oil seem have different function in sunflower development. The functional annotation of these PCgenes located within 100 kb from lncRNAs showing allelic bias were carried out by assignment of GO terms. For lncRNAs showing allelic bias toward cultivated lines for edible fruit, there were three enriched GO term including “cysteine-type endopeptidase activity”, “polysaccharide binding” and “pattern binding” (Additional file 11: Table S6). For lncRNAs showing allelic bias toward cultivated lines for oil, there were 15 enriched GO term including “ATP binding”, “carbohydrate derivative binding” and “pattern binding” and so on (Additional file 12: Table S7).

## Discussion

In recent years, growing evidence suggested that lncRNAs play an essential role in plant development and responses to stresses [54, 55]. So far, lncRNAs have been characterized in many plant species, such as Arabidopsis [13, 19], rice [21], maize [10] and wheat [21, 56]. Here, we undertook a genome-wide identification and characterization of lncRNAs and analyzed its methylation pattern in sunflower endosperm. In this study, 11,840 lncRNAs were identified by analyzing RNA-seq data of endosperm from two reciprocal crosses. The number of lncRNAs in sunflower endosperm is nearly twice more than that in caster bean [22]. The main reason may be the difference of genome size and complexity [57, 58]. Of course, the computational approaches prediction applied were different. Despite a large number of lncRNAs have been identified from many species, methods developed to date are not sufficiently accurate or comprehensive, which may cause incorrect and conflicting results [59]. In our study, we also found that the sequence lengths of lncRNAs are shorter, exon numbers are fewer, expression levels are lower, and have relatively specific tissue-specific expression when compared to PCgenes. These results are consistent with previous reports describing the common features in other plants [10, 21, 22, 60]. Also, we found that half of lncRNA tend to be expressed specifically in sunflower genotype. This implies that lncRNAs might share a common evolutionary pattern and have rapid turnover of lncRNA sequence.

LncRNAs can act in cis (neighboring genes) or in trans (distant genes) to regulate the expression of genes via transcriptional level, epigenetic modification level or post-transcriptional level [8, 61]. In previous work, about 20,000 lncRNA were identified in sunflower meiocytes [25]. And these lncRNAs potentially play roles in meiosis and may participate in the processes of chromatin modification [25]. In our study, a large number of lncRNAs were distant from PCgenes. Whether distant lncRNAs exert their function in trans, or as enhancers or insulators, needs to be further determined. A strong positive correlation was only present in a small number of lncRNA-PCgenes, suggesting that transcription of these genes may be coordinately regulated by adjacent lncRNA. It is tempting to speculate that coordinated transcription of lincRNAs with nearby PCgenes may be due to common regulatory sequences in their promoter regions, and/or that these lncRNAs themselves can positively regulate the transcription of nearby genes in cis. Seed oil content and quality is one of major breeding traits for sunflower [57]. We found that some genes homologous to Arabidopsis genes were metabolic pathways involved in oil synthesis and seed development (Additional file 13: Table S8). For example, we identified the lncNAT (HanXRQr2_lncRNA08192) located downstream of the gene HanXRQr2_Chr04g0171821, which was homologous to AT2G26640 (KCS11) in Arabidopsis, encoding KCS11, a putative member of the 3-ketoacyl-CoA synthase family involved in the biosynthesis of VLCFA (very long chain fatty acids). In eukaryotes, S-adenosylmethionine enzymes play roles in rRNA modifications [62, 63], tRNA modifications [64, 65], and lipid metabolism [66, 67]. The lncNAT (HanXRQr2_lncRNA09471) was expressed in downstream of the gene HanXRQr2_Chr06g0272911, which was homologous to AT4G13330 in Arabidopsis, encoding a putative S-adenosyl-L-methionine-dependent methyltransferases superfamily protein and may be related to fruit development. The target gene of lincRNA HanXRQr2_lncRNA00100 product is a putative FatA acyl-ACP thioesterase whose homologous gene in Arabidopsis is AT3G25110. Previous study showed that FatA is the dominant thioesterase during the period of oil accumulation in sunflower seeds [68]. Sunflower FatA acyl-ACP thioesterase is important not only for oil deposition in the seed but also, for the final oil composition [68]. These results suggested that lincRNA HanXRQr2_lncRNA00100 may regulate the expression of FatA, which could functions in the fatty acid biosynthesis pathway. Another lincRNA HanXRQr2_lncRNA02864 targets casein kinase I (CKI) gene, which encode a putative Ser/Thr kinase protein [69]. In rice, the activity of the lipase is controlled by the activity of riceCKI [70]. These may be involved in fatty acid biosynthesis pathway regulation. The protein-sequence homology of the target gene of lincRNA HanXRQr2_lncRNA09231 to Arabidopsis suggested that it encodes a putative Flavin-containing monooxygenase family protein (YUC10). In Arabidopsis, the YUC genes are mainly expressed in meristems, young primordia, vascular tissues, and reproductive organs, and it is essential for the formation of floral organs [71]. In maize, ZmYuc1 can affect endosperm development by regulating IAA biosynthesis [72]. These results suggested that lincRNA HanXRQr2_lncRNA09231 may be involved in seed development. The target gene of lincRNA HanXRQr2_lncRNA04387, which was homologous to AT4G00850 (GIF3) in Arabidopsis, encoding a putative GRF1-interacting factor 3. The GRF-INTERACTING FACTOR (GIF) family of Arabidopsis is an essential component required for the cell specification maintenance during reproductive organ development and, ultimately, for the reproductive competence [73]. This may imply that lincRNA HanXRQr2_lncRNA04387 is related to seed development. Transcription factors play important roles in plant development including floral organogenesis [74, 75], leaf initiation [76], lateral shoot initiation [77], gametogenesis [78] and seed development [79]. Those PCgenes showing strong correlation with the lncRNAs included 30 transcription factors (Additional file 14: Fig. S6). Hence, lncRNA potentially function in play roles in seed development. Along with the study of the coordinated transcription of lncRNA-PCgene pairs, additional mechanistic insights into the function of lncRNAs should be explored in future.

DNA methylation in plant have been focused on its regulation for gene expression [80]. In this study, we compared overall methylation levels between PCgenes and lncRNAs. We found that lncRNAs exhibited a much higher levels of DNA methylation than PCgenens, which might explain the low expression levels of lncRNAs. The similar expression pattern was also observed in castor bean [22]. Meanwhile, DNA methylation levels at transcription start sites were negatively correlated with lncRNAs expression levels, which was also the same with PCgenes. These finding indicate that DNA methylation may be related to regulation of lncRNAs expression in sunflower endosperm.

Genomic imprinting may be an important dosage control mechanism to regulate gene expression in a parent-of-origin-dependent manner [81]. Studies on the endosperm of rice, maize and castor bean identified a small number of imprinted lncRNAs [22, 33, 43]. Recently, a maternally expressed lncRNA MISSEN were reported as a regulator to modulate rice endosperm development [44]. Hence, identification and studies on the potential roles of imprinted lncRNAs in the triploid endosperm were meaningful for understanding the development of seed. In this study, we identified 36 imprinted lncRNAs by generating reciprocal crosses of different sunflower lines (Additional file 8: Table S4). Very similarly in rice and maize, the number of MNCs is significantly more than the number of PNCs [33, 43], suggesting that MNCs might have play more important roles in sunflower endosperm. In our study, we discovered most of imprinted lncRNAs showed parent-of-origin-dependent expression in certain genotypes but not in others. Major reason was due to lack of SNP. Hence, the density of SNPs was key limit for comparing the imprinting status of the lncRNAs in different reciprocal hybrids. Although the limited lncRNAs can be allelically analyzed in both of two crosses, imprinted lncRNAs show evidence of allelic variation for imprinting. However, how frequently imprinting variation of lncRNA is deserved to be research in future. The epigenetic profiles were also investigated for 36 imprinted non-coding RNAs. Result indicated the maternal demethylation at MNCs and the similar mechanism for epigenetic regulation of imprinted genes and non-coding RNAs.

In our study, 143 lncRNAs exhibiting allele-specific expression in cultivated sunflower lines for edible fruit and oil (Additional file 10: Table S5). Based on the result of GO analysis, we found lncRNAs showing allelic bias toward cultivated lines for edible fruit and cultivated lines for oil seem have different function in sunflower development. Serine carboxypeptidase (SCP) is a class of enzymes catalyzing proteolysis for functional protein maturation [82]. In rice, serine carboxypeptidase 46 has been reported to regulate grain filling [82]. lncRNAs showing allelic bias toward cultivated lines for oil are enriched in pathways related to serine-type peptidase activity. These results suggest that these lncRNAs may play a key role in grain filling in cultivated sunflower lines for oil. In peanut, differentially expressed genes in seed of different oil content varieties was analyzed for significant enrichment of GO terms [83]. Higher expression of generation of energy and metabolites was observed in peanut cv. Hanoch (high oil genotype) than 53 (low oil genotype) during seed development [83]. In grain filling in cultivated sunflower lines for oil, processes involving the generation of precursor metabolites and energy (e.g. ATP binding, adenyl nucleotide binding, carbohydrate derivative binding, oxidoreductase activity, pyrophosphatase activity) was significant enrichment (Additional file 12: Table S7). The result is similar to that reported in peanut. This might explain the differences in cultivated sunflower lines for edible fruit and oil.

## Conclusions

We comprehensively identified and analyzed11,840 lncRNAs in sunflower endosperm. Base on genome-wide analyses we found that the lncRNAs were relatively short, had fewer exons and a very tightly controlled tissue-specific expression compared to PCgenes. And a small fraction of lncRNAs exhibited coordinated expression with nearby PCgenes. Moreover, Genomic DNA methylation analyses revealed that the expression level of lncRNAs was tightly linked to DNA methylation. We further characterized expressed imprinted lncRNA during hybridization. Importantly, these results provide valuable information pointing to potential roles for lncRNAs in the development of sunflower endosperm. Our findings also shed light on the inheritance patterns of lncRNA expression and the epigenetic regulation of lncRNA itself in plants.

## Materials and methods

### Data sources

The datasets in this study were obtained from NCBI (https://www.ncbi.nlm.nih.gov) BioProject PRJNA740059 [51]. The RNA-seq datasets, YS2 endosperm (SRR14885491), SY2 endosperm (SRR14885492), YS1 endosperm (SRR14885493), SY1 endosperm (SRR14885498), were used for filtering potential lncRNAs. YS1(SRR14885497). The DNA methylation datasets, YA1(398A) embryo (SRR14885495), SY1 ensosperm (SRR14885496), YS1 ensosperm (SRR14885497), were used to analyze the average methylation levels for lncRNAs.

### Identification of lncRNAs and expression analysis

All raw reads containing adapter and low-quality reads were remove to obtain clean reads via Trim Galore (https://github.com/FelixKrueger/TrimGalore). The clean reads were used to align to reference genome of sunflower (https:// www.ncbi.nlm.nih.gov/assembly/GCF_ 00212 7325.2/), using HISAT2 [84]. After mapping to the reference genome of sunflower, the final transcriptome was assembled and quantified using StringTie [85].

After assembling and obtaining the transcripts, the process of lncRNA identification was based on their characteristics. The class-code of transcripts with ‘j’, ‘i’, ‘x’, ‘u’, ‘o’ and ‘e’ were chosen with Gffcompare for further analysis [85]. Then, we screened out the transcripts with length longer than 200 bp. Because lncRNA does not code protein, except the indictor of length and type, the transcript also should be evaluated whether it possessed the capability of coding protein. Based on the CPC2 (Coding Potential Calculator 2, identified label was ‘nocoding’) [86], CNCI (Coding-Non-Coding Index, identified label was ‘nocoding’) [87], PLEK (the Predictor of Long noncoding RNAs and mEssenger RNAs based on an improved K-mer scheme, identified label was ‘nocoding’) [88] and Pfam (E-value < 0.001) [89] analysis, the transcripts that could potentially code for a protein were removed. According to the FPKM values, transcripts that were less than 0.5 were discarded. The identified lncRNAs were further classified into four types of lncRNA by the genomic locations relative to PCgenes.

### Target gene prediction and functional annotation

To explore the function of lncRNAs in sunflower endosperm, we predicted the target genes of lncRNAs. In this study, PCgenes in 100 kb up- and downstream from the lncRNA, were selected by bedtools [24, 90]. To further function analysis, we identified a set of transcript pairs between the lincRNAs and the PCgenes transcribed within a 100 kb upstream or downstream of lincRNAs [91], and between the lncNATs and the corresponding PCgenes [22]. And the correlation in expression was evaluated using Pearson’s correlation coefficient (|r_p_| > 0.8 and *p* < 0.01) [22]. Pearson’s correlation coefficient and two-tailed Student’s t-test were calculated.

GO annotation was performed by InterProScan. The GO term enrichment analysis was conducted for genes included in each cluster using website (https://www.genescloud.cn/chart/GOenrich). All PCgenes and lncRNA-associated PCgenes were divided into two groups. GO categories among molecular function and biological process that show significant (*p* < 0.01) enrichment were displayed.

### Analysis of DNA methylation of lncRNA

DNA methylation data from the endosperm (SY1 and YS1) and embryo (398A) at 12 DAP were used to analyze the average methylation levels for lncRNAs, and the methylation ratios of CG, CHG and CHH sequence contexts were calculated as described in our previous study [51]. The methylation profiles in the 2-kb flanking regions and the lncRNA bodies were plotted based on the average methylation level for each 100-bp interval.

### Identification of imprinted lncRNA in sunflower 12 DAP endosperm

The SNP calling were performed as previously described [51]. according to the information of SNPs, we can divide the short sequences aligned at the SNP site from maternal or paternal allele. A series of Perl programs were used to calculate read counts from maternal or paternal allele at each SNPs. For a lncRNA, the number of reads that mapped to each allele was summed across all SNPs. Only transcripts that had at least 10 reads that could be assigned to a particular allele in each direction of the reciprocal cross could be analyzed. lncRNAs sites with significant bias (greater than or less than 2:1) in both hybrid endosperm tissues were considered as potentially imprinted lncRNAs. To obtain a subset of high-confidence imprinted lncRNAs, the favorable alleles were at least five times more than those of non-favorable alleles in both directions of a reciprocal cross, similar to the standard used in our previous study [51].

## Supplementary Information

**Additional file 1: Table S1.** The summary of sequencing data.

**Additional file 2: Table S2.** The genomic information of lncRNAs in sunflower endosperm at 12DAP.

**Additional file 3: Table S3.** GO Gene Ontology analysis of co-expressed protein-coding genes with all candidate lncRNAs.

**Additional file 4: Fig. S1.** Expressed lncRNAs in two crosses.

**Additional file 5: Fig. S2.** Expression levels of lncRNAs and PCgenes in sunflower endosperm.

**Additional file 6: Fig. S3.** DNA methylation profiles of long non-coding RNAs (lncRNAs) and protein-coding genes (PCgenes) in sunflower endosperm from SY1.

**Additional file 7: Fig. S4.** DNA methylation profiles of protein-coding genes (PCgenes) in sunflower endosperm from SY1.

**Additional file 8: Table S4.** The summary of imprinted lncRNA identified in 12 DAP sunflower endosperm.

**Additional file 9: Fig. S5.** Identification of imprinted long non-coding RNAs (lncRNAs) in sunflower endosperm at 12 DAP.

**Additional file 10: Table S5.** The summary of lncRNAs exhibiting allele-specific expression in cultivated sunflower lines for edible fruit and oil identified in 12 DAP sunflower endosperm.

**Additional file 11: Table S6.** GO Gene Ontology analysis of co-expressed protein-coding genes with lncRNAs of allelic bias toward cultivated lines for edible fruit.

**Additional file 12: Table S7.** GO Gene Ontology analysis of co-expressed protein-coding genes with lncRNAs of allelic bias toward cultivated lines for oil.

**Additional file 13: Table S8.** The annotation and homologs in Arabidopsis of these genes showing strong correlation with the lncRNAs were summarized in sunflower.

**Additional file 14: Fig. S6.** The number of transcription factors showing strong correlation with the lncRNAs.

**Additional file 15.** GTF File of candidate lncRNAs identified in sunflower endosperm.

## Data Availability

Sequencing datasets produced or investigated in this study are freely available at NCBI (PRJNA740059). The gene structure annotation file (in GTF format) of the lncRNAs is provided in Additional file 15.

## References

[1] Kim ED, Sung S (2012). Long noncoding RNA: unveiling hidden layer of gene regulatory networks. Trends Plant Sci.

[2] Hangauer MJ, Vaughn IW, McManus MT (2013). Pervasive transcription of the human genome produces thousands of previously unidentified long intergenic noncoding RNAs. PLoS Genet.

[3] Djebali S, Davis CA, Merkel A, Dobin A, Lassmann T, Mortazavi A, Tanzer A, Lagarde J, Lin W, Schlesinger F (2012). Landscape of transcription in human cells. Nature.

[4] Rahmioglu N, Nyholt DR, Morris AP, Missmer SA, Montgomery GW, Zondervan KT (2014). Genetic variants underlying risk of endometriosis: insights from meta-analysis of eight genome-wide association and replication datasets. Hum Reprod Update.

[5] Sharma S, Taneja M, Tyagi S, Singh K, Upadhyay SK (2017). Survey of high throughput RNA-Seq data reveals potential roles for lncRNAs during development and stress response in bread wheat. Front Plant Sci.

[6] Shi X, Sun M, Liu H, Yao Y, Song Y (2013). Long non-coding RNAs: a new frontier in the study of human diseases. Cancer Lett.

[7] Derrien T, Johnson R, Bussotti G, Tanzer A, Djebali S, Tilgner H, Guernec G, Martin D, Merkel A, Knowles DG (2012). The GENCODE v7 catalog of human long noncoding RNAs: analysis of their gene structure, evolution, and expression. Genome Res.

[8] Rinn JL, Chang HY (2012). Genome regulation by long noncoding RNAs. Annu Rev Biochem.

[9] Marques AC, Ponting CP (2009). Catalogues of mammalian long noncoding RNAs: modest conservation and incompleteness. Genome Biol.

[10] Li L, Eichten SR, Shimizu R, Petsch K, Yeh CT, Wu W, Chettoor AM, Givan SA, Cole RA, Fowler JE (2014). Genome-wide discovery and characterization of maize long non-coding RNAs. Genome Biol.

[11] Necsulea A, Soumillon M, Warnefors M, Liechti A, Daish T, Zeller U, Baker JC, Grützner F, Kaessmann H (2014). The evolution of lncRNA repertoires and expression patterns in tetrapods. Nature.

[12] Cabili MN, Trapnell C, Goff L, Koziol M, Tazon-Vega B, Regev A, Rinn JL (2011). Integrative annotation of human large intergenic noncoding RNAs reveals global properties and specific subclasses. Genes Dev.

[13] Liu J, Jung C, Xu J, Wang H, Deng S, Bernad L, Arenas-Huertero C, Chua NH (2012). Genome-wide analysis uncovers regulation of long intergenic noncoding RNAs in Arabidopsis. Plant Cell.

[14] Wang M, Yuan D, Tu L, Gao W, He Y, Hu H, Wang P, Liu N, Lindsey K, Zhang X (2015). Long noncoding RNAs and their proposed functions in fibre development of cotton (Gossypium spp.). New Phytol.

[15] Heo JB, Sung S (2011). Vernalization-mediated epigenetic silencing by a long intronic noncoding RNA. Science.

[16] Swiezewski S, Liu F, Magusin A, Dean C (2009). Cold-induced silencing by long antisense transcripts of an Arabidopsis Polycomb target. Nature.

[17] Ding J, Lu Q, Ouyang Y, Mao H, Zhang P, Yao J, Xu C, Li X, Xiao J, Zhang Q (2012). A long noncoding RNA regulates photoperiod-sensitive male sterility, an essential component of hybrid rice. Proc Natl Acad Sci U S A.

[18] Zhang L, Wang M, Li N, Wang H, Qiu P, Pei L, Xu Z, Wang T, Gao E, Liu J (2018). Long noncoding RNAs involve in resistance to Verticillium dahliae, a fungal disease in cotton. Plant Biotechnol J.

[19] Wang H, Chung PJ, Liu J, Jang IC, Kean MJ, Xu J, Chua NH (2014). Genome-wide identification of long noncoding natural antisense transcripts and their responses to light in Arabidopsis. Genome Res.

[20] Zhou X, Sunkar R, Jin H, Zhu JK, Zhang W (2009). Genome-wide identification and analysis of small RNAs originated from natural antisense transcripts in Oryza sativa. Genome Res.

[21] Zhang YC, Liao JY, Li ZY, Yu Y, Zhang JP, Li QF, Qu LH, Shu WS, Chen YQ (2014). Genome-wide screening and functional analysis identify a large number of long noncoding RNAs involved in the sexual reproduction of rice. Genome Biol.

[22] Xu W, Yang T, Wang B, Han B, Zhou H, Wang Y, Li DZ, Liu A (2018). Differential expression networks and inheritance patterns of long non-coding RNAs in castor bean seeds. Plant J.

[23] Wang M, Zhao W, Gao L, Zhao L (2018). Genome-wide profiling of long non-coding RNAs from tomato and a comparison with mRNAs associated with the regulation of fruit ripening. BMC Plant Biol.

[24] Ma X, Zhang X, Traore SM, Xin Z, Ning L, Li K, Zhao K, Li Z, He G, Yin D (2020). Genome-wide identification and analysis of long noncoding RNAs (lncRNAs) during seed development in peanut (Arachis hypogaea L.). BMC Plant Biol.

[25] Flórez-Zapata NM, Reyes-Valdés MH, Martínez O (2016). Long non-coding RNAs are major contributors to transcriptome changes in sunflower meiocytes with different recombination rates. BMC Genomics.

[26] Engreitz JM, Ollikainen N, Guttman M (2016). Long non-coding RNAs: spatial amplifiers that control nuclear structure and gene expression. Nat Rev Mol Cell Biol.

[27] Quinn JJ, Chang HY (2016). Unique features of long non-coding RNA biogenesis and function. Nat Rev Genet.

[28] Marchese FP, Raimondi I, Huarte M (2017). The multidimensional mechanisms of long noncoding RNA function. Genome Biol.

[29] Zhao X, Li J, Lian B, Gu H, Li Y, Qi Y (2018). Global identification of Arabidopsis lncRNAs reveals the regulation of MAF4 by a natural antisense RNA. Nat Commun.

[30] Li W, Chen Y, Wang Y, Zhao J, Wang Y (2022). Gypsy retrotransposon-derived maize lncRNA GARR2 modulates gibberellin response. Plant J.

[31] Yu Y, Zhou YF, Feng YZ, He H, Lian JP, Yang YW, Lei MQ, Zhang YC, Chen YQ (2020). Transcriptional landscape of pathogen-responsive lncRNAs in rice unveils the role of ALEX1 in jasmonate pathway and disease resistance. Plant Biotechnol J.

[32] Kim ED, Xiong Y, Pyo Y, Kim DH, Kang BH, Sung S (2017). Spatio-temporal analysis of coding and long noncoding transcripts during maize endosperm development. Sci Rep.

[33] Zhang M, Zhao H, Xie S, Chen J, Xu Y, Wang K, Zhao H, Guan H, Hu X, Jiao Y (2011). Extensive, clustered parental imprinting of protein-coding and noncoding RNAs in developing maize endosperm. Proc Natl Acad Sci U S A.

[34] Zhu M, Zhang M, Xing L, Li W, Jiang H, Wang L, Xu M (2017). Transcriptomic Analysis of Long Non-Coding RNAs and Coding Genes Uncovers a Complex Regulatory Network That Is Involved in Maize Seed Development. Genes (Basel).

[35] Shen E, Zhu X, Hua S, Chen H, Ye C, Zhou L, Liu Q, Zhu QH, Fan L, Chen X (2018). Genome-wide identification of oil biosynthesis-related long non-coding RNAs in allopolyploid Brassica napus. BMC Genomics.

[36] Yin DD, Li SS, Shu QY, Gu ZY, Wu Q, Feng CY, Xu WZ, Wang LS (2018). Identification of microRNAs and long non-coding RNAs involved in fatty acid biosynthesis in tree peony seeds. Gene.

[37] Das A, Nigam D, Junaid A, Tribhuvan KU, Kumar K, Durgesh K, Singh NK, Gaikwad K (2019). Expressivity of the key genes associated with seed and pod development is highly regulated via lncRNAs and miRNAs in Pigeonpea. Sci Rep.

[38] Jiang H, Jia Z, Liu S, Zhao B, Li W, Jin B, Wang L (2019). Identification and characterization of long non-coding RNAs involved in embryo development of Ginkgo biloba. Plant Signal Behav.

[39] Zhao J, Ajadi AA, Wang Y, Tong X, Wang H, Tang L, et al. Genome-wide identification of lncRNAs during Rice seed development. Genes (Basel). 2020;11(3):243. 10.3390/genes11030243 PMC7140839 32110990

[40] Coughlan JM, Wilson Brown M, Willis JH (2020). Patterns of hybrid seed Inviability in the Mimulus guttatus sp. Complex reveal a potential role of parental conflict in reproductive isolation.

[41] Kermicle JL (1970). Dependence of the R-mottled aleurone phenotype in maize on mode of sexual transmission. Genetics.

[42] Huh JH, Bauer MJ, Hsieh TF, Fischer RL (2008). Cellular programming of plant gene imprinting. Cell.

[43] Luo M, Taylor JM, Spriggs A, Zhang H, Wu X, Russell S, Singh M, Koltunow A (2011). A genome-wide survey of imprinted genes in rice seeds reveals imprinting primarily occurs in the endosperm. PLoS Genet.

[44] Zhou YF, Zhang YC, Sun YM, Yu Y, Lei MQ, Yang YW, Lian JP, Feng YZ, Zhang Z, Yang L (2021). The parent-of-origin lncRNA MISSEN regulates rice endosperm development. Nat Commun.

[45] Sreenivasulu N, Wobus U (2013). Seed-development programs: a systems biology-based comparison between dicots and monocots. Annu Rev Plant Biol.

[46] Zhang X, Yazaki J, Sundaresan A, Cokus S, Chan SW, Chen H, Henderson IR, Shinn P, Pellegrini M, Jacobsen SE (2006). Genome-wide high-resolution mapping and functional analysis of DNA methylation in arabidopsis. Cell.

[47] Yang H, Chang F, You C, Cui J, Zhu G, Wang L, Zheng Y, Qi J, Ma H (2015). Whole-genome DNA methylation patterns and complex associations with gene structure and expression during flower development in Arabidopsis. Plant J.

[48] Xing MQ, Zhang YJ, Zhou SR, Hu WY, Wu XT, Ye YJ, Wu XX, Xiao YP, Li X, Xue HW (2015). Global analysis reveals the crucial roles of DNA methylation during Rice seed development. Plant Physiol.

[49] He L, Huang H, Bradai M, Zhao C, You Y, Ma J, Zhao L, Lozano-Durán R, Zhu JK (2022). DNA methylation-free Arabidopsis reveals crucial roles of DNA methylation in regulating gene expression and development. Nat Commun.

[50] Pecrix Y, Buendia L, Penouilh-Suzette C, Maréchaux M, Legrand L, Bouchez O, Rengel D, Gouzy J, Cottret L, Vear F (2019). Sunflower resistance to multiple downy mildew pathotypes revealed by recognition of conserved effectors of the oomycete Plasmopara halstedii. Plant J.

[51] Zhang Z, Yu S, Li J, Zhu Y, Jiang S, Xia H, Zhou Y, Sun D, Liu M, Li C (2021). Epigenetic modifications potentially controlling the allelic expression of imprinted genes in sunflower endosperm. BMC Plant Biol.

[52] Edwards CA, Ferguson-Smith AC (2007). Mechanisms regulating imprinted genes in clusters. Curr Opin Cell Biol.

[53] Li E, Beard C, Jaenisch R (1993). Role for DNA methylation in genomic imprinting. Nature.

[54] Yu Y, Zhang Y, Chen X, Chen Y (2019). Plant noncoding RNAs: hidden players in development and stress responses. Annu Rev Cell Dev Biol.

[55] Ma X, Zhao F, Zhou B. The characters of non-coding RNAs and their biological roles in plant development and abiotic stress response. Int J Mol Sci. 2022;23(8):4124. 10.3390/ijms23084124 PMC9032736 35456943

[56] Cao P, Fan W, Li P, Hu Y (2021). Genome-wide profiling of long noncoding RNAs involved in wheat spike development. BMC Genomics.

[57] Badouin H, Gouzy J, Grassa CJ, Murat F, Staton SE, Cottret L, Lelandais-Brière C, Owens GL, Carrère S, Mayjonade B (2017). The sunflower genome provides insights into oil metabolism, flowering and Asterid evolution. Nature.

[58] Chan AP, Crabtree J, Zhao Q, Lorenzi H, Orvis J, Puiu D, Melake-Berhan A, Jones KM, Redman J, Chen G (2010). Draft genome sequence of the oilseed species Ricinus communis. Nat Biotechnol.

[59] Budak H, Kaya SB, Cagirici HB (2020). Long non-coding RNA in plants in the era of reference sequences. Front Plant Sci.

[60] Liu J, Li J, Liu HF, Fan SH, Singh S, Zhou XR, Hu ZY, Wang HZ, Hua W (2018). Genome-wide screening and analysis of imprinted genes in rapeseed (Brassica napus L.) endosperm. DNA Res.

[61] Faghihi MA, Wahlestedt C (2009). Regulatory roles of natural antisense transcripts. Nat Rev Mol Cell Biol.

[62] Yan F, LaMarre JM, Röhrich R, Wiesner J, Jomaa H, Mankin AS, Fujimori DG (2010). RlmN and Cfr are radical SAM enzymes involved in methylation of ribosomal RNA. J Am Chem Soc.

[63] Kaminska KH, Purta E, Hansen LH, Bujnicki JM, Vester B, Long KS (2010). Insights into the structure, function and evolution of the radical-SAM 23S rRNA methyltransferase Cfr that confers antibiotic resistance in bacteria. Nucleic Acids Res.

[64] Pierrel F, Douki T, Fontecave M, Atta M (2004). MiaB protein is a bifunctional radical-S-adenosylmethionine enzyme involved in thiolation and methylation of tRNA. J Biol Chem.

[65] Hernández HL, Pierrel F, Elleingand E, García-Serres R, Huynh BH, Johnson MK, Fontecave M, Atta M (2007). MiaB, a bifunctional radical-S-adenosylmethionine enzyme involved in the thiolation and methylation of tRNA, contains two essential [4Fe-4S] clusters. Biochemistry.

[66] Duschene KS, Broderick JB (2010). The antiviral protein viperin is a radical SAM enzyme. FEBS Lett.

[67] Hinson ER, Cresswell P (2009). The antiviral protein, viperin, localizes to lipid droplets via its N-terminal amphipathic alpha-helix. Proc Natl Acad Sci U S A.

[68] Aznar-Moreno JA, Sánchez R, Gidda SK, Martínez-Force E, Moreno-Pérez AJ, Venegas Calerón M, Garcés R, Mullen RT, Salas JJ (2018). New insights into sunflower (Helianthus annuus L.) FatA and FatB Thioesterases, their regulation, structure and distribution. Front Plant Sci.

[69] Su Y, Wang S, Zhang F, Zheng H, Liu Y, Huang T, Ding Y (2017). Phosphorylation of histone H2A at serine 95: a plant-specific mark involved in flowering time regulation and H2A.Z deposition. Plant Cell.

[70] Park HH (2012). Casein kinase I-like protein linked to lipase in plant. Plant Signal Behav.

[71] Cheng Y, Dai X, Zhao Y (2006). Auxin biosynthesis by the YUCCA flavin monooxygenases controls the formation of floral organs and vascular tissues in Arabidopsis. Genes Dev.

[72] Bernardi J, Lanubile A, Li QB, Kumar D, Kladnik A, Cook SD, Ross JJ, Marocco A, Chourey PS (2012). Impaired auxin biosynthesis in the defective endosperm18 mutant is due to mutational loss of expression in the ZmYuc1 gene encoding endosperm-specific YUCCA1 protein in maize. Plant Physiol.

[73] Lee BH, Wynn AN, Franks RG, Hwang YS, Lim J, Kim JH (2014). The Arabidopsis thaliana GRF-INTERACTING FACTOR gene family plays an essential role in control of male and female reproductive development. Dev Biol.

[74] Wang D, Oses-Prieto JA, Li KH, Fernandes JF, Burlingame AL, Walbot V (2010). The male sterile 8 mutation of maize disrupts the temporal progression of the transcriptome and results in the mis-regulation of metabolic functions. Plant J.

[75] Wang D, Chen X, Zhang Z, Liu D, Song G, Kong X, Geng S, Yang J, Wang B, Wu L (2015). A MADS-box gene NtSVP regulates pedicel elongation by directly suppressing a KNAT1-like KNOX gene NtBPL in tobacco (Nicotiana tabacum L.). J Exp Bot.

[76] Takatsuji H (1998). Zinc-finger transcription factors in plants. Cell Mol Life Sci.

[77] Takatsuji H (1999). Zinc-finger proteins: the classical zinc finger emerges in contemporary plant science. Plant Mol Biol.

[78] Kobayashi A, Sakamoto A, Kubo K, Rybka Z, Kanno Y, Takatsuji H (1998). Seven zinc-finger transcription factors are expressed sequentially during the development of anthers in petunia. Plant J.

[79] Zhang X, Zhao J, Wu X, Hu G, Fan S, Ma Q (2021). Evolutionary relationships and divergence of KNOTTED1-like family genes involved in salt tolerance and development in cotton (Gossypium hirsutum L.). Plant Sci.

[80] Wang W, Qin Q, Sun F, Wang Y, Xu D, Li Z, Fu B (2016). Genome-wide differences in DNA methylation changes in two contrasting Rice genotypes in response to drought conditions. Front Plant Sci.

[81] Yang G, Liu Z, Gao L, Yu K, Feng M, Yao Y, Peng H, Hu Z, Sun Q, Ni Z (2018). Genomic imprinting was evolutionarily conserved during wheat Polyploidization. Plant Cell.

[82] Li Z, Tang L, Qiu J, Zhang W, Wang Y, Tong X, Wei X, Hou Y, Zhang J (2016). Serine carboxypeptidase 46 regulates grain filling and seed germination in Rice (Oryza sativa L.). PLoS One.

[83] Gupta K, Kayam G, Faigenboim-Doron A, Clevenger J, Ozias-Akins P, Hovav R (2016). Gene expression profiling during seed-filling process in peanut with emphasis on oil biosynthesis networks. Plant Sci.

[84] Kim D, Langmead B, Salzberg SL (2015). HISAT: a fast spliced aligner with low memory requirements. Nat Methods.

[85] Pertea M, Pertea GM, Antonescu CM, Chang TC, Mendell JT, Salzberg SL (2015). StringTie enables improved reconstruction of a transcriptome from RNA-seq reads. Nat Biotechnol.

[86] Kang YJ, Yang DC, Kong L, Hou M, Meng YQ, Wei L, Gao G (2017). CPC2: a fast and accurate coding potential calculator based on sequence intrinsic features. Nucleic Acids Res.

[87] Sun L, Luo H, Bu D, Zhao G, Yu K, Zhang C, Liu Y, Chen R, Zhao Y (2013). Utilizing sequence intrinsic composition to classify protein-coding and long non-coding transcripts. Nucleic Acids Res.

[88] Li A, Zhang J, Zhou Z (2014). PLEK: a tool for predicting long non-coding RNAs and messenger RNAs based on an improved k-mer scheme. BMC Bioinformatics.

[89] Finn RD, Bateman A, Clements J, Coggill P, Eberhardt RY, Eddy SR, Heger A, Hetherington K, Holm L, Mistry J (2014). Pfam: the protein families database. Nucleic Acids Res.

[90] Quinlan AR, Hall IM (2010). BEDTools: a flexible suite of utilities for comparing genomic features. Bioinformatics.

[91] Liao Q, Liu C, Yuan X, Kang S, Miao R, Xiao H, Zhao G, Luo H, Bu D, Zhao H (2011). Large-scale prediction of long non-coding RNA functions in a coding-non-coding gene co-expression network. Nucleic Acids Res.

