# Supplementary material for: Genome-wide identification and characterization of lncRNAs in sunflower endosperm
Source: BMC Plant Biol. 2022 Oct 22;22:494. doi: 10.1186/s12870-022-03882-5 (PMC9587605; doi:10.1186/s12870-022-03882-5)
Supplement: Supplementary file 1 — Additional file 1: Table S1. The summary of sequencing data. [file 12870_2022_3882_MOESM1_ESM.docx]

**Table S1. The summary of sequencing data.**

| **Sequencing** | **Sample** | **Run Type** | **Raw Reads** | **Clean Reads** | **Alignment Rate** |
| --- | --- | --- | --- | --- | --- |
| **RNA-seq** | 12 DAP Endosperm(SY1) | 150 bp Pair end | 46,025,144 | 45,373,368 | 88.90% |
|  | 12 DAP Endosperm(YS1) |  | 45,517,182 | 44,953,138 | 89.70% |
|  | 12 DAP Endosperm(SY2) |  | 46,098,422 | 45,258,640 | 91.06% |
|  | 12 DAP Endosperm(YS2) |  | 48,279,256 | 47,051,462 | 90.40% |
