# Supplementary material for: Genome-wide identification and characterization of lncRNAs in sunflower endosperm
Source: BMC Plant Biol. 2022 Oct 22;22:494. doi: 10.1186/s12870-022-03882-5 (PMC9587605; doi:10.1186/s12870-022-03882-5)
Supplement: Supplementary file 4 — Additional file 4: Fig. S1. Expressed lncRNAs in two crosses. [file 12870_2022_3882_MOESM4_ESM.docx]

**
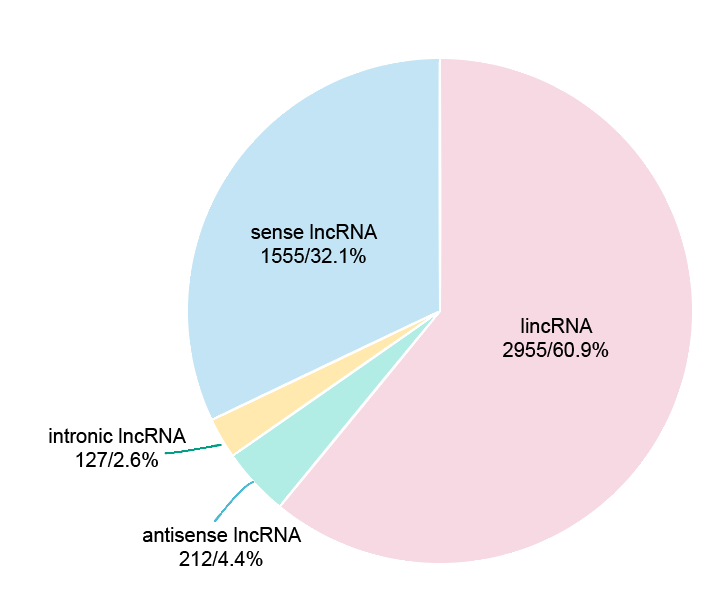
**

**Fig. S1. Expressed lncRNAs in two crosses.**

The composition of different types of the number of common lncRNAs in the four libraries.
