# Supplementary material for: Genome-wide identification and characterization of lncRNAs in sunflower endosperm
Source: BMC Plant Biol. 2022 Oct 22;22:494. doi: 10.1186/s12870-022-03882-5 (PMC9587605; doi:10.1186/s12870-022-03882-5)
Supplement: Supplementary file 5 — Additional file 5: Fig. S2. Expression levels of lncRNAs and PCgenes in sunflower endosperm. [file 12870_2022_3882_MOESM5_ESM.docx]

**
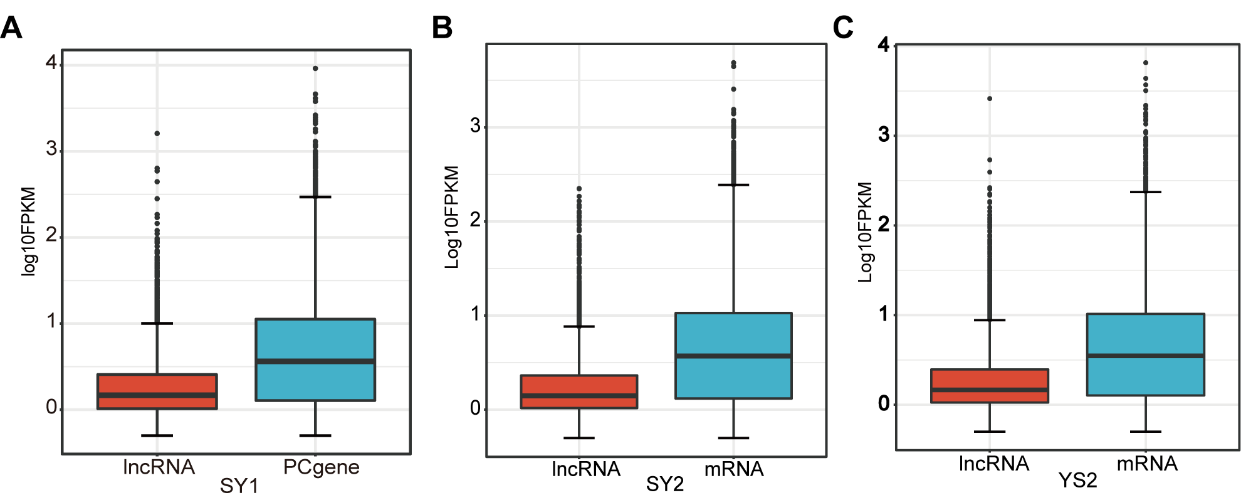
**

**Fig. S2. Expression levels of lncRNAs and PCgenes in sunflower endosperm.**

Expression levels of lncRNAs and PCgenes in SY1 endosperm (A), SY2 endosperm (B) and YS2 endosperm (C) as illustrated by the boxplot.
