# Supplementary material for: Genome-wide identification and characterization of lncRNAs in sunflower endosperm
Source: BMC Plant Biol. 2022 Oct 22;22:494. doi: 10.1186/s12870-022-03882-5 (PMC9587605; doi:10.1186/s12870-022-03882-5)
Supplement: Supplementary file 7 — Additional file 7: Fig. S4. DNA methylation profiles of protein-coding genes (PCgenes) in sunflower endosperm from SY1. [file 12870_2022_3882_MOESM7_ESM.docx]

**
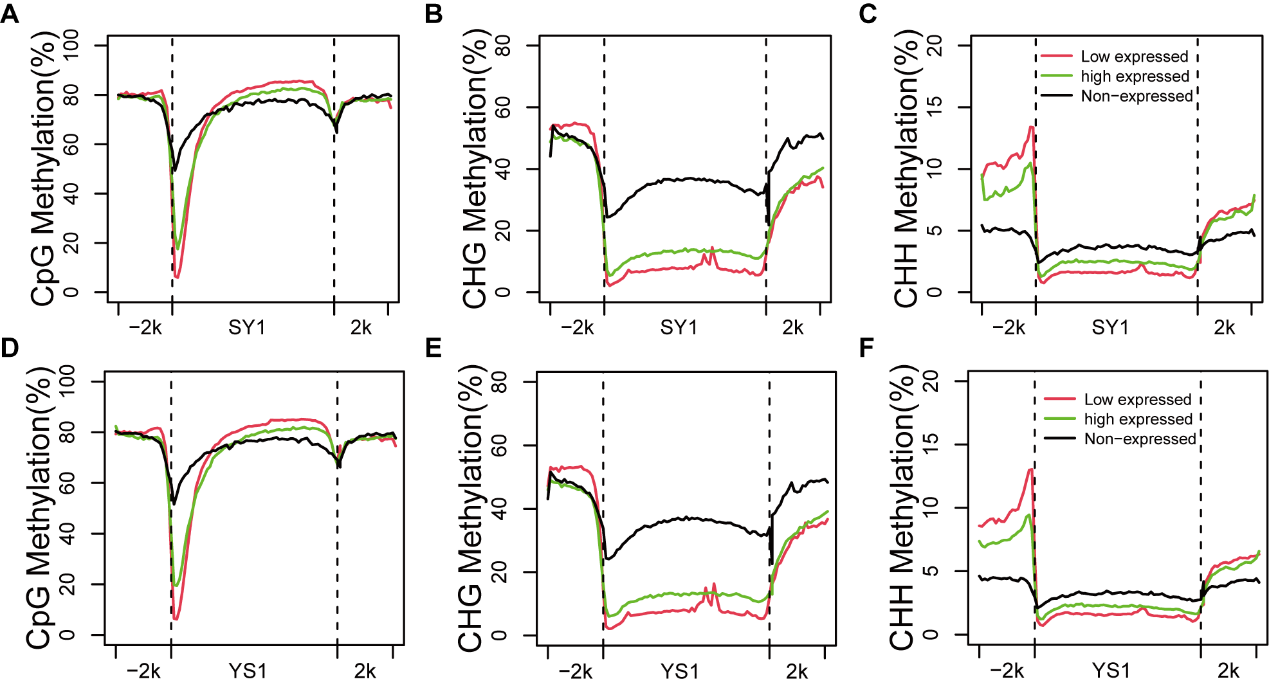
**

**Fig. S4. DNA methylation profiles of protein-coding genes (PCgenes) in sunflower endosperm from SY1.**

(A-C) Association between DNA methylation and PCgenes expression in CG, CHG and CHH sequence contexts throughout the gene body and its 2-kb up- and downstream regions in SY1 endosperm; (D-F) Association between DNA methylation and PCgenes expression in CG, CHG and CHH sequence contexts throughout the gene body and its 2-kb up- and downstream regions in YS1 endosperm.
