# Supplementary material for: Genome-wide identification and characterization of lncRNAs in sunflower endosperm
Source: BMC Plant Biol. 2022 Oct 22;22:494. doi: 10.1186/s12870-022-03882-5 (PMC9587605; doi:10.1186/s12870-022-03882-5)
Supplement: Supplementary file 9 — Additional file 9: Fig. S5. Identification of imprinted long non-coding RNAs (lncRNAs) in sunflower endosperm at 12 DAP. [file 12870_2022_3882_MOESM9_ESM.docx]

**
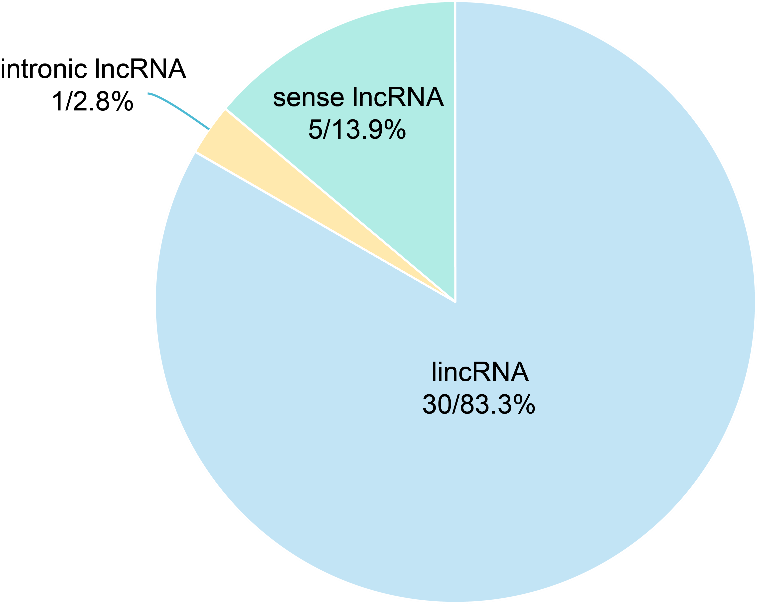
**

**Fig. S5. Identification of imprinted long non-coding RNAs (lncRNAs) in sunflower endosperm at 12 DAP.**

Classification of total identified imprinted lncRNAs including lincRNA, antisense-lncRNA, intronic-lncRNA and sense-lncRNA.
