# Supplementary material for: Genome-wide identification and characterization of lncRNAs in sunflower endosperm
Source: BMC Plant Biol. 2022 Oct 22;22:494. doi: 10.1186/s12870-022-03882-5 (PMC9587605; doi:10.1186/s12870-022-03882-5)
Supplement: Supplementary file 14 — Additional file 14: Fig. S6. The number of transcription factors showing strong correlation with the lncRNAs. [file 12870_2022_3882_MOESM14_ESM.docx]

**Fig. S6. The number of transcription factors showing strong correlation with the lncRNAs.**
